# Unraveling SNAP: distinct patterns of early neurodegeneration through MRI texture analysis

**DOI:** 10.1016/j.nicl.2025.103829

**Published:** 2025-06-18

**Authors:** Min Jeong Kwon, Jieun Park, Sungman Jo, Jun Sung Kim, Hyukjun Lee, Dae Jong Oh, Ji Won Han, Ki Woong Kim

**Affiliations:** a Department of Brain and Cognitive Science, Seoul National University College of Natural Science, Seoul, Republic of Korea; b Department of Health Science and Technology, Graduate School of Convergence Science and Technology, Seoul National University, Seoul, Republic of Korea; c Department of Neuropsychiatry, Seoul National University Bundang Hospital, Sungnam, Republic of Korea; d Workplace Mental Health Institute, Kangbuk Samsung Hospital, Sungkyunkwan University School of Medicine, Seoul, Republic of Korea; e Department of Psychiatry, Seoul National University College of Medicine, Seoul, Republic of Korea; f Institute of Human Behavioral Medicine, Seoul National University Medical Research Center, Seoul, Republic of Korea

**Keywords:** SNAP (suspected non-alzheimer disease pathophysiology), Texture, Volume, MRI, Neurodegeneration

## Abstract

**Aim:**

Suspected non-Alzheimer’s disease pathophysiology (SNAP) is a condition characterized by neurodegeneration in the absence of amyloid beta (Aβ) deposition, posing challenges for early diagnosis. This study aimed to investigate the progression of neurodegeneration in SNAP through the analysis of brain MRI volume and texture.

**Methods:**

The study included 449 amyloid-negative participants categorized into three groups: cognitively normal without neurodegeneration (N-CN), cognitively normal with neurodegeneration (N + CN), and MCI with neurodegeneration (N + MCI). Volume and texture metrics were derived from T1-weighted MRI. Texture analysis quantified microstructural changes using grey level co-occurrence matrices, while volume metrics measured atrophy.

**Results:**

Texture changes were observed earlier and more widely than volume reductions. In N + CN, texture changes were present in the hippocampus, entorhinal cortex and orbitofrontal cortex. In N + MCI, texture changes extended to frontal and subcortical regions, including the thalamus and putamen, while volume reductions extended to the lateral temporal cortex and amygdala.

**Conclusions:**

Texture analysis is a sensitive tool for detecting early neurodegenerative changes in SNAP, capturing microstructural changes preceding volume loss. By integrating texture and volume metrics, this study highlights a distinct neurodegenerative trajectory in SNAP. Future research should validate these findings longitudinally and explore the clinical application of texture metrics.

## Highlights

• Texture changes in SNAP appear more widely than volume reductions.
• MRI texture analysis supports early detection of SNAP before atrophy occurs.
• Texture and volume metrics offer insights into the neurodegenerative pattern of SNAP.

## 1 Introduction

Suspected non-Alzheimer disease pathophysiology (SNAP) is a condition characterized by the presence of abnormal levels of neurodegeneration markers (N+) in the absence of amyloid beta (Aβ) markers. (Jack et al., 2018) Despite its prevalence − affecting approximately 23 % of older adults with normal cognition and 25 % of those with mild cognitive impairment (MCI) − SNAP remains underdiagnosed due to a lack of standardized biomarkers and diagnostic protocols. Unlike Alzheimer’s disease (AD), which benefits from advanced diagnostic tools such as amyloid PET imaging and cerebrospinal fluid (CSF) analyses, (Janelidze et al., 2016, Klunk et al., 2004) the heterogeneous etiology of SNAP complicates its identification. Pathological processes underlying SNAP include argyrophilic grain disease, α-synucleinopathy, and TDP-43 proteinopathy. (Jack et al., 2016) which diverge from the amyloid-driven mechanisms observed in AD. This variability underscores the need for innovative diagnostic approaches to improve understanding, detection, and management of SNAP.

Early diagnosis of SNAP is critical for timely intervention and differentiation from AD, as their clinical presentations often overlap. Structural magnetic resonance imaging (MRI) is widely used to assess neurodegeneration, but conventional volumetric measures primarily capture macroscopic atrophy, limiting their sensitivity to early microstructural changes. This poses a significant challenge in identifying SNAP in its preclinical stages, where neurodegeneration may be diffuse but subtle. Individuals with SNAP have worse clinical outcomes than Aβ-negative individuals without neurodegeneration markers (N-). (Jack et al., 2016) While volumetric measures are effective in detecting gross structural loss in advanced stages, they often fail to detect the nuanced changes that precede overt atrophy.

Texture analysis has emerged as a promising neuroimaging technique to address these limitations. Unlike volumetric analysis, texture analysis quantifies variations in voxel intensity and spatial distribution, providing insight into microstructural tissue integrity. Previous studies have shown that texture changes often precede volumetric changes in neurodegenerative diseases, highlighting their potential as early biomarkers. (Lee et al., 2020, Moon et al., 2023) For example, in AD, texture analysis has been shown to predict progression to dementia earlier than hippocampal atrophy. (Lee et al., 2020) Texture analysis is particularly well-suited to the study of SNAP because it captures spatial heterogeneity in brain tissue that may arise from diffuse, microstructural changes such as gliosis, synaptic loss, or small vessel disease. These pathologies are often seen in non-AD neurodegenerative conditions and may not produce visible atrophy in early stages. Therefore, texture metrics offer a unique advantage in detecting early, amyloid-independent neurodegeneration. This sensitivity to early pathological changes suggests that texture analysis could enhance the detection of SNAP and provide critical insights into its distinct neurodegenerative trajectory.

This study aims to characterize the progression patterns of neurodegeneration in SNAP using texture and volume metrics derived from structural MRI. By integrating complementary imaging approaches, this study aims to advance the understanding of the unique pathophysiology of SNAP and contribute to the development of accurate diagnostic tools that can inform targeted therapeutic strategies.

## 2 Methods

### 2.1 Participants

The study included 449 community-dwelling older adults aged 60 years or older, all of whom were confirmed amyloid-negative on 18F-florbetaben PET imaging. Amyloid negativity was defined as a brain amyloid plaque load (BAPL) score of less than grade 2, as determined by expert neuroradiologists.

Participants were recruited from two sources: 235 individuals attending the dementia clinic at Seoul National University Bundang Hospital (SNUBH) and 214 participants in the Korean Longitudinal Study on Cognitive Aging and Dementia (KLOSCAD). The KLOSCAD cohort is a nationwide, prospective study designed to assess cognitive ageing and dementia in a representative sample of 6,818 older Koreans. Participants were assessed every two years from 2010 to 2020. (Han et al., 2018).

Eligibility criteria included the absence of major psychiatric disorders (e.g., mood or substance use disorders), neurological disorders (e.g., cerebrovascular disease, epilepsy), and other medical conditions that could significantly affect cognition, as well as cerebral infarction, severe white matter hyperintensities (WMH) on the Fazekas scale (grade = 3), or cerebral hemorrhages on brain MRI.

### 2.2 Ethical considerations

All participants gave written informed consent and the study protocol was approved by the Institutional Review Board of SNUBH (IRB No. B-2005-615-001). All procedures adhered to the tenets of the Declaration of Helsinki.

### 2.3 Diagnostic assessment

Standardized diagnostic assessments were performed by geriatric neuropsychiatrists, including review of medical history, physical and neurological examinations, and assessments using the Korean version of the Consortium to Establish a Registry for Alzheimer's Disease (CERAD-K) Clinical Assessment Battery (Lee et al., 2002) and the Korean version of the Mini International Neuropsychiatric Interview. (Yoo et al., 2006) Neuropsychological testing was performed by research neuropsychologists or trained nurses using the CERAD-K Neuropsychological Assessment Battery, which consists of nine tests: Verbal Fluency, Boston Naming, Mini-Mental State Examination (MMSE), Word List Memory, Word List Recall, Word List Recognition, Constructional Practice, Constructional Recall, and Trail Making Test A/B. (Lee et al., 2002).

A panel of geriatric psychiatrists determined the final diagnoses and Clinical Dementia Rating (CDR) (Morris, 1993) scores for all participants. Dementia and major psychiatric disorders were diagnosed according to the Diagnostic and Statistical Manual of Mental Disorders, Fourth Edition (DSM-IV) (Bell, 1994) and mild cognitive impairment (MCI) according to the consensus criteria of the International Working Group on MCI. (Winblad et al., 2004).

As pathological changes in SNAP may begin before clinical symptoms become pronounced in SNAP, as in AD, (Douglas & Scharre, 2019) this study included participants with preclinical (cognitively normal) and prodromal (MCI) stages to capture the early stages of SNAP,. Participants were stratified into three diagnostic groups based on cognitive function and neurodegeneration markers derived from medial temporal atrophy (MTA) grades on T1-weighted MRI: (1) cognitively normal without neurodegeneration (N-CN, n = 183), (2) cognitively normal with neurodegeneration (N + CN, n = 111), and (3) MCI with neurodegeneration (N + MCI, n = 155). The presence of neurodegeneration (N + ) was defined as grade 2 or higher MTA on coronal slices of T1-weighted brain MRI according to the Scheltens scale. (Scheltens et al., 1992) An MTA score of ≥ 2 has been commonly used as a threshold for abnormal atrophy in individuals under 75 years of age. (Cavallin et al., 2012, Westman et al., 2011) MTA ratings were performed independently by two neuroradiologists who were blinded to diagnostic group. The inter-rater reliability was excellent, with an ICC of 0.924 (95 % CI: 0.899–0.942) for the left hemisphere and 0.859 (95 % CI: 0.816–0.893) for the right hemisphere. The distribution of MTA scores across diagnostic groups is provided in Supplementary Table 1 to support the validity of this criterion.

### 2.4 MRI acquisition and preprocessing

All MRI scans, including those from both sources (the SNUBH clinic and KLOSCAD), were acquired at Seoul National University Bundang Hospital (SNUBH) using the same 3.0 T Philips Achieva scanner and identical acquisition protocols. Specifically, three-dimensional (3D) T1-weighted spoiled gradient-echo images were obtained with the following parameters: echo time = 4.6 ms, repetition time = 8.1 ms, flip angle = 8°, voxel size = 1.0 × 0.5 × 0.5 mm^3^, and sagittal slice thickness = 1.0 mm with no interslice gap. The original images were converted from Digital Imaging and Communications in Medicine (DICOM) to Neuroimaging Informatics Technology Initiative (NIfTI) format and resliced into isovoxels (1.0 × 1.0 × 1.0 mm^3^). Brain structures were parcellated using FreeSurfer 6.0 (http://surfer.nmr.mgh.harvard.edu),(Fischl et al., 2002) following the Desikan-Killiany-Tourville (DKT) atlas.(Klein & Tourville, 2012) Pre-processing steps included bias field correction to reduce intensity non-uniformity(Ashburner & Friston, 2005), motion correction, skull stripping and segmentation into anatomically defined regions of interest (ROIs).

### 2.5 Volume and texture analysis

Brain volumes were calculated as the sum of voxel intensities within each ROI, normalized to total brain volume (TBV) to control for inter-individual differences. We used TBV as the sum of the volumes of all structures identified in the aseg.mgz file by the *recon-all* function in FreeSurfer version 6.0 (http://surfer.nmr.mgh.harvard.edu). (Fischl et al., 2002).

Texture analysis was performed on pre-processed T1-weighted images. For histogram normalization, the partial volume effect was corrected by including voxels with intensity values between [μ − 3σ] and [μ + 3σ] only (μ, mean; σ, standard deviation). (Collewet et al., 2004) Signal intensities within each ROI were normalized with respect to the participant’s mean cerebrospinal fluid (CSF) signal intensity in the lateral ventricles to correct for inter-individual differences. Subsequently, voxel intensities were re-quantized to 32 grey levels to reduce statistical sparsity, as recommended in prior studies, (Orlhac et al., 2018) (Zhang, 2012) in order to balance texture detail preservation and computational efficiency. (Patel et al., 2008) Texture features were extracted using a 3D grey level co-occurrence matrix (GLCM) approach implemented in MATLAB R2021a (MathWorks, Natick, MA, USA). For each ROI, texture metrics, including contrast, were calculated to quantify voxel intensity variation. Texture contrast reflects microstructural heterogeneity, with higher values indicating increased grey level variation. Analyses were performed at a voxel distance of 1 across 13 spatial directions, with results averaged to provide robust texture estimates for each ROI. The GLCM is an N × N matrix, where N represents the total number of grey levels present within the image. The matrix element (i,j) denotes the frequency of specific grey level pairs, including the reference voxel i and the neighboring voxel j, occurring at distance d and direction θ. 3D GLCMs were generated at a distance of d = 1 from each other (directly adjacent voxels) in 13 different directions. Based on the averaged 13 GLCMs, the “contrast” in each region was calculated using Haralick texture features (Haralick et al., 1973). The contrast texture feature measures local grey-level variation in an image, reflecting both the spatial distribution and the relative difference in gray- levels of adjacent voxels. Specifically, contrast increases as the difference in grey-levels between adjacent voxel pairs increase, enabling the simplest and most intuitive interpretation of texture changes. The formula for calculating contrast is shown below.

$$\text{Contrast}\;\sum_{1=1}^{N}\sum_{j=1}^{N}{\left(i-j\right)}^{2}P_{i.j}=$$

*where*

N, the number of distinct gray levels in the quantized image.

P_i,j_, (i,j)th entry in a normalized gray-level co-occurrence matrix.

### 2.6 Statistical analysis

Demographic and clinical variables were compared between groups using one-way analysis of variance (ANOVA) for continuous variables and chi-squared tests for categorical variables. Regional brain volumes and texture metrics were analysed using analysis of covariance (ANCOVA), adjusting for age, sex, years of education, and TBV (for volume analyses) or regional volume (for texture analyses). For each ROI, Bonferroni correction (p < 0.05/3) was applied to post hoc comparisons among the three diagnostic groups. The p-values reported in the tables correspond to Bonferroni-corrected values for within-ROI comparisons. To complement significance testing, effect sizes were calculated using partial eta squared (η^2^).

All statistical analyses were performed using SPSS version 25.0 (IBM Corporation; Armonk, NY, USA) and MedCalc version 18.11.3 (MedCalc Software, Mariakerke, Belgium), with two-tailed p-values < 0.05 considered statistically significant.

## 3 Results

The three groups did not differ significantly in terms of age, gender or educational level. However, the N + MCI group had significantly lower MMSE scores than both the N-CN and N + CN groups (p < 0.001), indicating greater cognitive impairment (Table 1).

**Table 1:** Characteristics of the participants.

	N-CN^a^	N + CN^b^	N + MCI ^c^	Statistics*	
	(n = 183)	(n = 111)	(n = 155)	*p*	Post-hoc
Age, years, mean (SD)	74.4 (4.5)	75.3 (4.4)	74.8 (4.4)	0.224	
Sex, female, %	63.4	51.4	61.9	0.102	
Education, years, mean (SD)	12.0 (4.8)	12.1 (5.0)	10.9 (5.2)	0.061	
Total brain volume^†^, cc, mean (SD)	985.5 (86.8)	980.7 (91.5)	962.7 (91.2)	0.055	
MMSE, points, mean (SD)	27.6 (2.1)	26.9 (2.8)	24.1 (3.5)	< 0.001	a, b > c

N-CN, cognitively normal without neurodegeneration; N + CN, cognitively normal with neurodegeneration; N + MCI, mild cognitive impairment with neurodegeneration; MMSE, Mini-Mental State Examination

* One-way analysis of variance for continuous variables and chi-square test for categorical variables with Bonferroni post hoc comparisons.

Analysis of regional brain volumes revealed significant differences between diagnostic groups, particularly in the temporal lobe. The N + MCI group had significantly reduced volumes in temporal subregions including the insula, amygdala, hippocampus, entorhinal cortex, inferior temporal gyrus and middle temporal gyrus compared to the other groups (p < 0.005). In contrast, the N + CN group showed intermediate reductions, mainly in the hippocampus and entorhinal cortex, compared to the N-CN group (p < 0.01). No significant volume differences were observed in the parietal or occipital lobes between the groups, highlighting the relative sparing of these regions in early stages of SNAP (Table 2).

**Table 2:** Comparison of regional volumes between diagnostic groups.

	N-CN^a^	N + CN^b^	N + MCI ^c^	Statistics*			
	(n = 183)	(n = 111)	(n = 155)	F	*p*	effect size	post-hoc
**Temporal lobe**	105.9 (9.6)	105.9 (9.8)	100.9 (11.1)	15.573	<0.001	0.066	a, b > c
Insula	12.8 (1.3)	12.8 (1.3)	12.2 (1.3)	7.068	0.001	0.031	a, b > c
Amygdala	2.7 (0.3)	2.6 (0.4)	2.5 (0.5)	8.427	<0.001	0.037	a > c
Hippocampus	7.2 (0.7)	6.8 (0.7)	6.4 (0.9)	50.755	<0.001	0.187	a > b > c
Entorhinal cortex	3.8 (0.6)	3.8 (0.7)	3.4 (0.8)	16.517	<0.001	0.070	a, b > c
Para hippocampal	3.3 (0.4)	3.3 (0.4)	3.2 (0.5)	1.299	0.274	0.006	
Fusiform	16.3 (1.7)	16.3 (1.8)	15.8 (1.9)	1.850	0.158	0.008	
Bankssts	3.9 (0.5)	3.9 (0.6)	3.8 (0.6)	0.905	0.405	0.004	
Inferior temporal	19.2 (2.6)	19.1 (2.4)	18.1 (2.7)	7.516	0.001	0.033	a, b > c
Middle temporal	19.7 (2.4)	19.6 (2.4)	18.6 (2.5)	7.516	0.001	0.033	a, b > c
Superior temporal	20.2 (2.2)	20.5 (2.2)	19.7 (2.5)	2.972	0.052	0.014	
Transverse temporal	1.7 (0.3)	1.7 (0.3)	1.6 (0.3)	0.414	0.661	0.002	
Temporal pole	4.7 (0.5)	4.7 (0.5)	4.6 (0.7)	1.781	0.170	0.008	
**Frontal lobe**	151.1 (14.4)	152.1 (13.8)	149 (14.8)	1.969	0.141	0.009	
Orbitofrontal	21.9 (2.2)	22.1 (2.3)	21.4 (2.2)	1.022	0.361	0.005	
Inferior frontal	17.8 (2.2)	17.5 (1.9)	17.2 (1.9)	2.364	0.095	0.011	
Middle frontal	35.3 (4.1)	35.6 (4.1)	34.9 (4.4)	1.405	0.246	0.006	
Superior frontal	36.6 (3.8)	36.8 (3.7)	36.3 (3.9)	1.507	0.223	0.007	
Precentral	23.8 (2.4)	24.2 (2.6)	24.4 (2.7)	0.233	0.792	0.001	
Paracentral	6.8 (0.8)	6.8 (0.7)	6.7 (0.8)	1.195	0.304	0.005	
Frontal pole	1.9 (0.2)	1.9 (0.2)	1.8 (0.2)	0.359	0.699	0.002	
Anterior cingulate	6.9 (1.1)	6.9 (1.1)	6.7 (1.1)	0.459	0.632	0.002	
**Parietal lobe**	106.3 (9.7)	106.7 (10.6)	104.8 (10.8)	2.229	0.109	0.010	
Inferior parietal	22.9 (2.9)	22.6 (3.1)	22.3 (3.0)	0.036	0.965	0.000	
Superior parietal	22.6 (2.4)	23.0 (2.6)	23.2 (2.9)	0.335	0.716	0.002	
Postcentral	16.2 (1.8)	16.3 (1.9)	16.2 (1.9)	2.957	0.053	0.013	
Precuneus	16.7 (1.8)	16.8 (2.0)	16.3 (1.8)	0.230	0.100	0.010	
Supra marginal	18.2 (2.1)	18.3 (2.3)	17.9 (2.2)	0.393	0.675	0.002	
Isthmus cingulate	4.4 (0.6)	4.3 (0.5)	4.3 (0.6)	0.893	0.410	0.004	
Posterior cingulate	5.5 (0.7)	5.5 (0.7)	5.3 (0.9)	1.101	0.333	0.005	
**Occipital lobe**	40.3 (4.7)	40.4 (5)	40.0 (4.6)	0.877	0.417	0.004	
Cuneus	5.4 (0.8)	5.4 (0.8)	5.4 (0.8)	1.235	0.292	0.006	
Lingual	11.3 (1.4)	11.2 (1.6)	11.0 (1.5)	0.250	0.779	0.001	
Lateral occipital	19.8 (2.6)	20.0 (2.8)	19.8 (2.6)	2.052	0.130	0.009	
Pericalcarine	3.9 (0.7)	3.9 (0.7)	3.9 (0.7)	0.385	0.681	0.002	
**Subcortical gray matter**	30.7 (3.0)	30.6 (3.2)	30.2 (3.2)	0.095	0.909	0.000	
Accumbens area	0.8 (0.2)	0.8 (0.1)	0.8 (0.2)	2.471	0.086	0.011	
Caudate	6.5 (1.0)	6.7 (1.1)	6.5 (1.1)	2.395	0.092	0.011	
Putamen	8.4 (1.1)	8.2 (1.1)	8.2 (1.2)	0.828	0.438	0.004	
Pallidum	3.3 (0.4)	3.3 (0.5)	3.2 (0.4)	0.194	0.824	0.001	
Thalamus	11.7 (1.1)	11.6 (1.1)	11.5 (1.1)	0.449	0.639	0.002	
**Cerebellum**	117.9 (11.0)	117.7 (12.3)	117.0 (11.5)	1.433	0.240	0.006	

Note. All values are presented as mean (standard deviation) in cc.

N-CN, cognitively normal without neurodegeneration; N + CN, cognitively normal with neurodegeneration; N + MCI, mild cognitive impairment with neurodegeneration.

* One-way analysis of covariance adjusting for total brain volume, age, sex, and education. Bonferroni correction applied to post hoc comparisons within each ROI.

Texture analysis revealed widespread microstructural changes with significant group differences in temporal, frontal and subcortical regions. Both the N + CN and N + MCI groups showed higher texture contrast in temporal subregions compared to the N-CN group (p < 0.001). In particular, the N + MCI group showed the highest texture contrast in regions such as the entorhinal cortex, hippocampus and parahippocampal gyrus. In addition, significant texture differences were observed in frontal (orbitofrontal cortex, middle frontal cortex) and subcortical regions (putamen, thalamus) in the N + MCI group compared to the N-CN group (p < 0.01) **(**Table 3**)**. Post-hoc power estimates along with detailed statistical results are summarized in Supplementary Tables 2 and 3. Exploratory analyses using entropy and autocorrelation as alternative GLCM texture features also demonstrated similar patterns of group differences across key regions (Supplementary Tables 4 and 5). Comparisons of regional volume and texture patterns highlight the distinct progression of neurodegeneration in SNAP. While volume loss was largely confined to temporal regions, texture changes were more widespread and included temporal, frontal and subcortical areas. Texture contrast changes in the entorhinal cortex, hippocampus and putamen were particularly pronounced, suggesting early microstructural disorganization that may serve as a sensitive marker for the detection of SNAP.

**Table 3:** Comparison of regional textures between diagnostic groups.

	N-CN^a^	N + CN^b^	N + MCI ^c^	Statistics*			
	(n = 183)	(n = 111)	(n = 155)	F	*p*	effect size	post-hoc
**Temporal lobe**	18.8 (2.3)	19.7 (2.0)	20.4 (2.1)	11.818	<0.001	0.051	a < b, c
Insula	22.4 (1.9)	23.0 (1.8)	23.3 (1.8)	7.209	0.001	0.032	a < b, c
Amygdala	23.8 (3.1)	24.0 (3.1)	24.8 (3.2)	3.019	0.050	0.013	
Hippocampus	26.9 (2.0)	26.6 (2.4)	27.6 (2.2)	5.739	0.003	0.025	a, b < c
Entorhinal cortex	25.8 (2.9)	27.1 (3.6)	29.0 (4.1)	19.839	<0.001	0.082	a < b < c
Parahippocampus	26.9 (2.7)	27.6 (3)	28.9 (3.5)	15.329	<0.001	0.065	a < b, c
Fusiform	23.3 (2.7)	23.8 (2.2)	24.0 (2.5)	3.075	0.047	0.014	
Bankssts	24.4 (2.8)	25.3 (2.9)	25.3 (3.2)	3.347	0.036	0.015	
Inferior temporal	21.4 (2.2)	22.0 (2.0)	22.6 (2.2)	7.458	0.001	0.033	a < c
Middle temporal	20.3 (2.2)	21.0 (2.0)	21.5 (2.3)	6.256	0.002	0.028	a < b, c
Superior temporal	21.8 (2.0)	22.0 (1.6)	22.5 (1.7)	3.473	0.032	0.015	a < c
Transverse temporal	34.3 (5.0)	35.3 (5.3)	35.7 (5.2)	1.649	0.193	0.007	
Temporal pole	26.0 (2.0)	26.1 (2.8)	26.5 (2.7)	1.216	0.297	0.005	
**Frontal lobe**	18.4 (2.1)	18.8 (2.1)	18.2 (2.2)	2.214	0.110	0.010	
Orbitofrontal	23.2 (2.6)	23.9 (2.3)	23.3 (3.3)	3.163	0.043	0.014	
Inferior frontal	24.7 (2.1)	25.6 (1.7)	25.8 (2.0)	10.666	<0.001	0.046	a < b, c
Middle frontal	23.2 (2.0)	24.2 (1.8)	24.1 (2.0)	12.490	<0.001	0.053	a < b, c
Superior frontal	20.6 (2.2)	21.8 (2.1)	21.9 (2.1)	17.610	<0.001	0.074	a < b, c
Precentral	21.7 (2.0)	22.0 (2.0)	21.9 (2.3)	2.148	0.0.118	0.010	
Paracentral	26.4 (3.4)	27.2 (3.4)	26.9 (3.7)	0.935	0.394	0.004	
Frontal pole	28.5 (2.2)	28.9 (2.4)	29.0 (2.8)	2.005	0.136	0.009	
Anterior cingulate	23.6 (2.1)	24.2 (2.3)	24.0 (1.7)	2.370	0.095	0.011	
**Parietal lobe**	17.9 (1.7)	18.3 (1.6)	18.1 (1.8)	1.491	0.226	0.007	
Inferior parietal	23.7 (2.2)	24.5 (2.1)	24.4 (2.2)	3.336	0.036	0.015	a < b, c
Superior parietal	25.6 (2.5)	26.2 (2.8)	25.6 (2.5)	0.679	0.507	0.003	
Postcentral	25.2 (2.7)	25.5 (2.3)	25.1 (2.6)	0.330	0.719	0.001	
Precuneus	22.7 (3.0)	23.1 (3.3)	22.3 (3.1)	2.414	0.091	0.011	
Supra marginal	21.2 (1.9)	21.5 (1.7)	21.6 (1.9)	1.890	0.152	0.008	
Isthmus cingulate	24.0 (1.8)	24.7 (2.1)	24.3 (2.1)	1.839	0.160	0.008	
Posterior cingulate	24.9 (1.8)	25.5 (2.1)	25.7 (1.9)	5.568	0.004	0.025	a < b, c
**Occipital lobe**	24.6 (2.7)	24.8 (2.2)	24.8 (2.5)	0.196	0.822	0.001	
Cuneus	34.0 (4.8)	34.1 (4.9)	33.1 (4.8)	2.268	0.105	0.010	
Lingual	29.0 (3.2)	29.8 (3.5)	29.9 (3.4)	2.835	0.060	0.013	
Lateral occipital	28.1 (2.7)	28.7 (2.2)	28.6 (2.5)	2.014	0.135	0.009	
Pericalcarine	40.5 (5.9)	40.6 (5.1)	40.8 (5.4)	0.219	0.804	0.001	
**Subcortical gray matter**	12.8 (1.4)	13.0 (1.5)	13.0 (1.4)	1.023	0.360	0.005	
Accumbens area	27.7 (4.3)	27.4 (4.5)	26.5 (4.4)	2.944	0.054	0.013	
Caudate	20.6 (2.1)	21.5 (1.9)	21.4 (2.0)	9.438	<0.001	0.041	a < b, c
Putamen	23.4 (3.4)	24.4 (3.6)	25.4 (3.6)	11.326	<0.001	0.049	a < b, c
Pallidum	26.8 (3.8)	26.3 (3.9)	27.0 (3.9)	1.396	0.249	0.006	
Thalamus	15.0 (1.4)	15.5 (1.6)	15.5 (1.5)	4.590	0.011	0.020	a < b, c
**Cerebellum**	11.4 (1.2)	11.8 (1.5)	11.9 (1.5)	5.128	0.006	0.023	a < c

Note. All values are presented as mean (standard deviation).

N-CN, cognitively normal without neurodegeneration; N + CN, cognitively normal with neurodegeneration; N + MCI, mild cognitive impairment with neurodegeneration.

* One-way analysis of covariance adjusting for corresponding regional volume, age, sex, and education. Bonferroni correction applied to post hoc comparisons within each ROI.

**Fig. 1:**
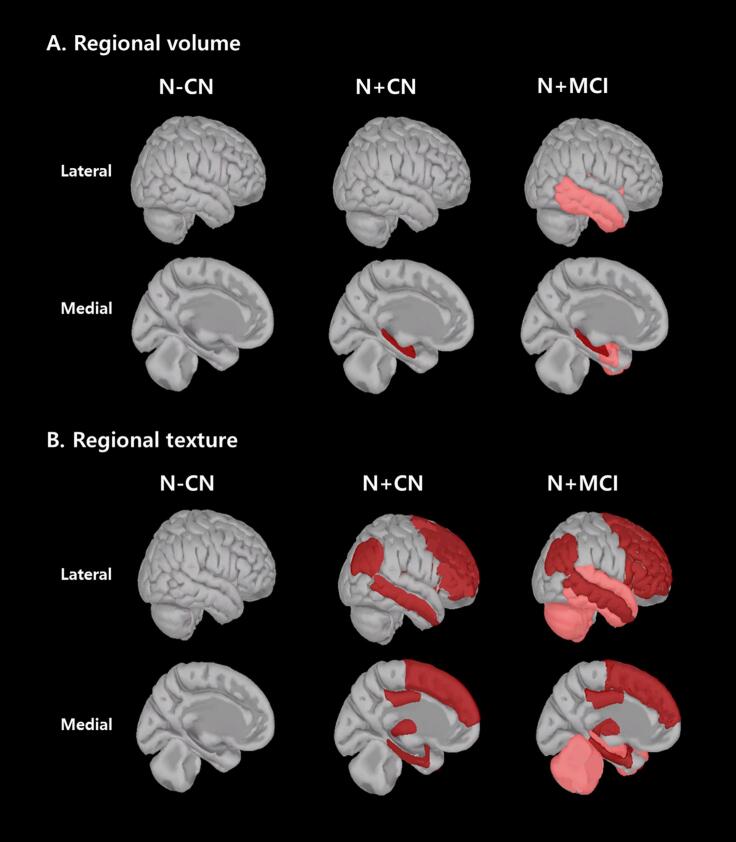
3d brain visualization of regional volume and texture changes according to stage progression **(A)** Regional volume changes in each diagnostic group **(B)** Regional texture changes in each diagnostic group Note. Dark red clusters represent regions with significant differences identified in post-hoc analysis between N-CN and N + CN. Light red clusters represent regions with significant differences identified in post-hoc analysis between N-CN and N + MCI. N-CN, cognitively normal without neurodegeneration; N + CN, cognitively normal with neurodegeneration; N + MCI, mild cognitive impairment with neurodegeneration. (For interpretation of the references to colour in this figure legend, the reader is referred to the web version of this article.)

Fig. 1 illustrates these findings in more detail, highlighting the sequential progression of neurodegeneration in SNAP. In the preclinical stage (N + CN), textural changes were evident in several regions, including the hippocampus, entorhinal cortex, parahippocampal gyrus and orbitofrontal cortex, even before significant volume reductions became apparent. In contrast, volume reductions at this stage were mainly confined to the hippocampus, suggesting that gross atrophy occurs later in the disease process. As SNAP progresses to the prodromal stage (N + MCI), volume reductions extend to additional temporal regions such as the lateral temporal cortex, insula and amygdala, while texture changes become more widespread. These include frontal regions (e.g. middle frontal and orbitofrontal cortices), subcortical areas (e.g. thalamus and putamen) and the cerebellum. Notably, the parietal and occipital lobes remained relatively spared in both volume and texture, highlighting a distinct pattern compared to AD. This sparing further supports the hypothesis that SNAP follows a unique pathological trajectory. The overlapping regions of texture and volume changes, such as the hippocampus and entorhinal cortex, highlight the sequential nature of these changes. These results summarize a distinct spatial pattern of texture and volume alterations across SNAP stages, with regional overlap most prominent in medial temporal areas.

## 4 Discussion

This study provides novel insights into the progression of neurodegeneration in SNAP using texture and volume metrics derived from structural MRI. Our results support the hypothesis that microstructural changes, as captured by texture analysis, may occur in the absence of volumetric reductions and could serve as an early, sensitive marker for the detection of SNAP. These findings have important implications for improving the early diagnosis and differentiation of SNAP from AD.

The study showed that textural changes were widespread and occurred earlier than volumetric changes, consistent with the hypothesis that microstructural disorganization precedes overt atrophy in SNAP. At the preclinical stage, texture changes were observed in regions such as the hippocampus, entorhinal cortex and orbitofrontal cortex, even in the absence of significant volume loss. This temporal dissociation highlights the unique strength of texture analysis in identifying the earliest disruptions in cellular and extracellular architecture. Taken together, these findings support the potential of microstructural disorganization as an early imaging marker of neurodegeneration in SNAP. These findings suggest that texture analysis captures subtle cellular and synaptic changes that may be driven by heterogeneous pathologies such as tauopathy, TDP-43 proteinopathy and α-synucleinopathy (Wisse et al., 2021).

Texture analysis has demonstrated its ability to detect early pathological changes that may be missed by traditional volumetric measures. Previous studies, including our own, have shown that texture metrics can serve as early biomarkers in a number of neurodegenerative diseases. For example, MRI texture analysis predicted progression from MCI to dementia in AD earlier than hippocampal volume loss (Lee et al., 2020). In addition, MRI texture metrics were found to correlate with specific radiographic pathologies in AD, further validating their utility as sensitive indicators of early neurodegenerative changes. (Lee et al., 2021) The distinction observed in MRI texture metrics of the medial pulvinar in dementia with Lewy bodies compared to controls with comparable volumes underscores the broader applicability of texture analysis across different neurodegenerative disorders. (Tak et al., 2020) These texture changes reflect subtle changes in neuronal density, myelin and other tissue characteristics that may not immediately result in visible atrophy, but are indicative of early neurodegeneration. While this study primarily focused on contrast as the main GLCM texture feature, additional findings from entropy and autocorrelation (Supplementary Tables 4 and 5) support that the observed texture alterations are not limited to a single descriptor. These findings highlight the potential of texture analysis to capture the earliest disruptions in microstructural integrity, making it a valuable tool for detecting preclinical neurodegeneration in SNAP. Importantly, our interpretation is also supported by prior longitudinal findings. For instance, a previous study (Sørensen et al., 2016) demonstrated that T1-derived texture features predicted future hippocampal atrophy and cognitive decline in MCI, suggesting that texture abnormalities can precede overt atrophy in certain neurodegenerative contexts. This sensitivity to early changes positions texture metrics as a critical complement to volumetric measures, particularly in conditions where traditional imaging fails to detect early pathological changes. Therefore, texture analysis may play a vital role in improving the early detection and characterization of SNAP-related neurodegeneration.

Our results highlight a distinct neurodegenerative trajectory in SNAP, characterized by early involvement of temporal and subcortical regions, with relative sparing of the parietal and occipital lobes. This pattern contrasts with AD, where parietal atrophy is a hallmark even in the early stages. (Bakkour et al., 2009, Guo et al., 2010) The progression of structural changes from temporal to frontal and subcortical regions, including the cerebellum, underscores the diffuse and heterogeneous nature of SNAP-related neurodegeneration. These observations are consistent with the hypothesis that SNAP represents a spectrum of non-amyloid pathologies with distinct pathways of progression, further emphasizing the need for diagnostic tools that can capture this complexity. Notably, the underlying pathologies associated with SNAP, such as α-synucleinopathy, tauopathy and TDP-43 proteinopathy, preferentially affect brain regions different from those commonly implicated in AD. For example, α-synuclein pathology is predominantly observed in the cerebellum, putamen, insula, and frontal and temporal cortices, as seen in Lewy body dementia and PD dementia.(Borghammer et al., 2010, Burton et al., 2002, Camicioli et al., 2009, Cousins et al., 2003, Reetz et al., 2009, Seidel et al., 2017) Tau pathology, often associated with frontotemporal dementia, affects the frontal lobe, anterior insula and thalamus,(Cairns et al., 2007, Davidson et al., 2007, Rohrer and Rosen, 2013) while TDP-43 proteinopathy affects the frontal and temporal cortices and hippocampus, (Cairns et al., 2007, Davidson et al., 2007, Rohrer and Rosen, 2013) with similar patterns observed in amyotrophic lateral sclerosis (Geser et al., 2009, Leigh et al., 1991, Neumann et al., 2006). These findings suggest that the pathological drivers of SNAP may induce neurodegeneration in regions that are relatively spared in AD, contributing to the distinct progression pattern observed in our study.

A key strength of this study is its methodological rigor, including the use of well-characterized participants with clearly defined diagnostic criteria and the integration of advanced imaging techniques. Importantly, all MRI scans were acquired on a single scanner using a standardized protocol, eliminating potential variability due to scanner differences. This enhances the internal validity of our imaging analyses. In addition, the use of amyloid PET imaging to exclude Aβ-positive individuals ensured that findings were specific to SNAP and not confounded by overlapping AD pathology. These methodological approaches strengthen the reliability and interpretability of the results, while the combination of texture and volume metrics adds a complementary dimension to traditional neuroimaging studies.

Despite its strengths, this study has several limitations. First, the cross-sectional design precludes examination of longitudinal changes and their temporal relationships with cognitive decline. Future studies should include longitudinal data to validate the temporal dissociation between texture and volume changes and to assess their predictive value for clinical outcomes. Such follow-up studies will be essential to determine the prognostic utility of texture features and to confirm whether early textural changes translate into meaningful clinical trajectories over time. Second, although texture analysis was effective in detecting early changes, the underlying biological correlates of these changes remain unclear. Integrating CSF or serum biomarkers in future studies may enhance the interpretability of texture findings and support a multimodal understanding of early neurodegenerative processes. Emerging plasma biomarkers such as p-tau217, neurofilament light chain (NfL), and α-synuclein offer promising avenues for non-invasive validation of microstructural alterations. Incorporating these markers may help to link in vivo texture changes with specific proteinopathies Additionally, combining imaging with histopathological validation may provide deeper insights into the mechanisms driving texture changes in SNAP. Third, the sample size, although sufficient for statistical analysis, may limit the generalizability of the findings to broader populations. Larger, multi-center studies are needed to confirm these findings and to explore potential subtypes within SNAP.

This study demonstrates that texture analysis is a sensitive and effective tool for detecting early neurodegenerative changes in SNAP, complementing traditional volumetric approaches. By capturing microstructural changes that precede volume loss, texture analysis provides critical insight into the distinct progression patterns of SNAP and its differentiation from AD. These findings highlight the potential of combining texture and volume metrics to deepen our understanding of the diverse pathology underlying SNAP. Future research should focus on validating these findings longitudinally and integrating them into clinical workflows to improve early diagnosis and patient outcomes.

## CRediT authorship contribution statement

**Min Jeong Kwon:** Writing – original draft, Methodology, Conceptualization. **Jieun Park:** Formal analysis. **Sungman Jo:** Formal analysis. **Jun Sung Kim:** Formal analysis. **Hyukjun Lee:** Data curation. **Dae Jong Oh:** Data curation. **Ji Won Han:** Writing – review & editing, Data curation. **Ki Woong Kim:** Writing – review & editing, Writing – original draft, Data curation, Conceptualization.

## Declaration of competing interest

The authors declare that they have no known competing financial interests or personal relationships that could have appeared to influence the work reported in this paper.

## Data Availability

Data will be made available on request.
