# Supplementary material for: Unraveling SNAP: distinct patterns of early neurodegeneration through MRI texture analysis
Source: Neuroimage Clin. 2025 Jun 18;47:103829. doi: 10.1016/j.nicl.2025.103829 (PMC12221672; doi:10.1016/j.nicl.2025.103829)
Supplement: Supplementary Data 1 [file mmc1.docx]

**Supplementary Table 1. Distribution of Medial Temporal Atrophy in participants**

|  | N-CN^a^ | N+CN^b^ | N+MCI ^c^ | *Statistics^*^* | |
| --- | --- | --- | --- | --- | --- |
|  | (n = 183) | (n = 111) | (n = 155) | *p* | Post-hoc |
| Left hemisphere , mean (SD) | 0.6 (0.5) | 1.6 (0.5) | 1.9 (0.5) | < 0.001 | a < b < c |
| Right hemisphere, mean (SD) | 0.6 (0.5) | 1.6 (0.5) | 1.9 (0.4) | < 0.001 | a < b < c |

N-CN, cognitively normal without amyloid beta deposition and neurodegeneration; N+CN, cognitively normal with neurodegeneration but not amyloid beta deposition; N+MCI, mild cognitive impairment with neurodegeneration but not amyloid beta deposition

^*^One-way analysis of variance with Bonferroni post hoc comparisons

**Supplementary Table 2. Summary of statistical results for regional volume comparisons among diagnostic groups**

|  |  | | | Statistics | | |
| --- | --- | --- | --- | --- | --- | --- |
|  |  | *p* | effect size | | post-hoc | post-hoc power |
| **Temporal lobe** |  | <0.001 | 0.066 | | a, b > c | 1.000 |
| Insula |  | 0.001 | 0.031 | | a, b > c | 0.933 |
| Amygdala |  | <0.001 | 0.037 | | a > c | 0.968 |
| Hippocampus |  | <0.001 | 0.187 | | a > b > c | 1.000 |
| Entorhinal cortex |  | <0.001 | 0.070 | | a, b > c | 1.000 |
| Para hippocampal |  | 0.274 | 0.006 | |  | 0.292 |
| Fusiform |  | 0.158 | 0.008 | |  | 0.378 |
| Bankssts |  | 0.405 | 0.004 | |  | 0.206 |
| Inferior temporal |  | 0.001 | 0.033 | | a, b > c | 0.947 |
| Middle temporal |  | 0.001 | 0.033 | | a, b > c | 0.947 |
| Superior temporal |  | 0.052 | 0.014 | |  | 0.609 |
| Transverse temporal |  | 0.661 | 0.002 | |  | 0.123 |
| Temporal pole |  | 0.170 | 0.008 | |  | 0.378 |
| **Frontal lobe** |  | 0.141 | 0.009 | |  | 0.420 |
| Orbitofrontal |  | 0.361 | 0.005 | |  | 0.249 |
| Inferior frontal |  | 0.095 | 0.011 | |  | 0.500 |
| Middle frontal |  | 0.246 | 0.006 | |  | 0.292 |
| Superior frontal |  | 0.223 | 0.007 | |  | 0.335 |
| Precentral |  | 0.792 | 0.001 | |  | 0.085 |
| Paracentral |  | 0.304 | 0.005 | |  | 0.249 |
| Frontal pole |  | 0.699 | 0.002 | |  | 0.123 |
| Anterior cingulate |  | 0.632 | 0.002 | |  | 0.123 |
| **Parietal lobe** |  | 0.109 | 0.010 | |  | 0.461 |
| Inferior parietal |  | 0.965 | 0.000 | |  | 0.000 |
| Superior parietal |  | 0.716 | 0.002 | |  | 0.123 |
| Postcentral |  | 0.053 | 0.013 | |  | 0.574 |
| Precuneus |  | 0.100 | 0.010 | |  | 0.461 |
| Supra marginal |  | 0.675 | 0.002 | |  | 0.123 |
| Isthmus cingulate |  | 0.410 | 0.004 | |  | 0.206 |
| Posterior cingulate |  | 0.333 | 0.005 | |  | 0.249 |
| **Occipital lobe** |  | 0.417 | 0.004 | |  | 0.206 |
| Cuneus |  | 0.292 | 0.006 | |  | 0.292 |
| Lingual |  | 0.779 | 0.001 | |  | 0.085 |
| Lateral occipital |  | 0.130 | 0.009 | |  | 0.420 |
| Pericalcarine |  | 0.681 | 0.002 | |  | 0.123 |
| **Subcortical gray matter** |  | 0.909 | 0.000 | |  | 0.000 |
| Accumbens area |  | 0.086 | 0.011 | |  | 0.500 |
| Caudate |  | 0.092 | 0.011 | |  | 0.500 |
| Putamen |  | 0.438 | 0.004 | |  | 0.206 |
| Pallidum |  | 0.824 | 0.001 | |  | 0.085 |
| Thalamus |  | 0.639 | 0.002 | |  | 0.123 |
| **Cerebellum** |  | 0.240 | 0.006 | |  | 0.292 |

Note. Power was estimated from observed effect sizes using one-way ANOVA (α = 0.05)

**Supplementary Table 3. Summary of statistical results for regional textures comparisons among diagnostic groups**

|  |  | | | Statistics | | |
| --- | --- | --- | --- | --- | --- | --- |
|  |  | *p* | effect size | | post-hoc | post-hoc power |
| **Temporal lobe** |  | <0.001 | 0.051 | | a < b, c | 0.995 |
| Insula |  | 0.001 | 0.032 | | a < b, c | 0.940 |
| Amygdala |  | 0.050 | 0.013 | |  | 0.574 |
| Hippocampus |  | 0.003 | 0.025 | | a, b < c | 0.867 |
| Entorhinal cortex |  | <0.001 | 0.082 | | a < b < c | 1.000 |
| Parahippocampus |  | <0.001 | 0.065 | | a < b, c | 0.999 |
| Fusiform |  | 0.047 | 0.014 | |  | 0.609 |
| Bankssts |  | 0.036 | 0.015 | |  | 0.641 |
| Inferior temporal |  | 0.001 | 0.033 | | a < c | 0.947 |
| Middle temporal |  | 0.002 | 0.028 | | a < b, c | 0.905 |
| Superior temporal |  | 0.032 | 0.015 | | a < c | 0.641 |
| Transverse temporal |  | 0.193 | 0.007 | |  | 0.335 |
| Temporal pole |  | 0.297 | 0.005 | |  | 0.249 |
| **Frontal lobe** |  | 0.110 | 0.010 | |  | 0.461 |
| Orbitofrontal |  | 0.043 | 0.014 | |  | 0.609 |
| Inferior frontal |  | <0.001 | 0.046 | | a < b, c | 0.990 |
| Middle frontal |  | <0.001 | 0.053 | | a < b, c | 0.996 |
| Superior frontal |  | <0.001 | 0.074 | | a < b, c | 1.000 |
| Precentral |  | .0.118 | 0.010 | |  | 0.461 |
| Paracentral |  | 0.394 | 0.004 | |  | 0.206 |
| Frontal pole |  | 0.136 | 0.009 | |  | 0.420 |
| Anterior cingulate |  | 0.095 | 0.011 | |  | 0.500 |
| **Parietal lobe** |  | 0.226 | 0.007 | |  | 0.335 |
| Inferior parietal |  | 0.036 | 0.015 | | a < b, c | 0.641 |
| Superior parietal |  | 0.507 | 0.003 | |  | 0.164 |
| Postcentral |  | 0.719 | 0.001 | |  | 0.085 |
| Precuneus |  | 0.091 | 0.011 | |  | 0.500 |
| Supra marginal |  | 0.152 | 0.008 | |  | 0.378 |
| Isthmus cingulate |  | 0.160 | 0.008 | |  | 0.378 |
| Posterior cingulate |  | 0.004 | 0.025 | | a < b, c | 0.867 |
| **Occipital lobe** |  | 0.822 | 0.001 | |  | 0.085 |
| Cuneus |  | 0.105 | 0.010 | |  | 0.461 |
| Lingual |  | 0.060 | 0.013 | |  | 0.574 |
| Lateral occipital |  | 0.135 | 0.009 | |  | 0.420 |
| Pericalcarine |  | 0.804 | 0.001 | |  | 0.085 |
| **Subcortical gray matter** |  | 0.360 | 0.005 | |  | 0.249 |
| Accumbens area |  | 0.054 | 0.013 | |  | 0.574 |
| Caudate |  | <0.001 | 0.041 | | a < b, c | 0.981 |
| Putamen |  | <0.001 | 0.049 | | a < b, c | 0.994 |
| Pallidum |  | 0.249 | 0.006 | |  | 0.292 |
| Thalamus |  | 0.011 | 0.020 | | a < b, c | 0.776 |
| **Cerebellum** |  | 0.006 | 0.023 | | a < c | 0.835 |

Note. Power was estimated from observed effect sizes using one-way ANOVA (α = 0.05)

**Supplementary Table 4. Comparison of regional GLCM entropy across diagnostic groups**

|  | N-CN^a^ | N+CN^b^ | N+MCI ^c^ | Statistics^*^ | | | |
| --- | --- | --- | --- | --- | --- | --- | --- |
|  | (n = 183) | (n = 111) | (n = 155) | F | *p* | effect size | post-hoc |
| **Temporal lobe** | 5.68 (0.06) | 5.70 (0.05) | 5.72 (0.06) | 12.896 | <0.001 | 0.055 | a < b, c |
| Insula | 5.66 (0.07) | 5.66 (0.06) | 5.68 (0.06) | 2.241 | 0.108 | 0.010 |  |
| Amygdala | 5.56 (0.13) | 5.52 (0.12) | 5.53 (0.13) | 2.395 | 0.092 | 0.011 |  |
| Hippocampus | 5.64 (0.08) | 5.61 (0.09) | 5.67 (0.07) | 10.785 | <0.001 | 0.047 | a, b < c |
| Entorhinal cortex | 5.72 (0.07) | 5.74 (0.06) | 5.76 (0.07) | 14.443 | <0.001 | 0.061 | a < b, c |
| Parahippocampus | 5.71 (0.06) | 5.73 (0.06) | 5.75 (0.06) | 17.425 | <0.001 | 0.073 | a < b, c |
| Fusiform | 5.79 (0.06) | 5.80 (0.04) | 5.81 (0.05) | 2.570 | 0.078 | 0.011 |  |
| Bankssts | 5.65 (0.06) | 5.66 (0.06) | 5.65 (0.07) | 0.363 | 0.695 | 0.002 |  |
| Inferior temporal | 5.71 (0.06) | 5.72 (0.05) | 5.74 (0.05) | 12.062 | <0.001 | 0.052 | a, b < c |
| Middle temporal | 5.63 (0.06) | 5.64 (0.05) | 5.65 (0.07) | 4.263 | 0.015 | 0.019 | a < c |
| Superior temporal | 5.64 (0.06) | 5.64 (0.05) | 5.66 (0.06) | 3.091 | 0.046 | 0.014 |  |
| Transverse temporal | 5.66 (0.10) | 5.66 (0.09) | 5.66 (0.09) | 0.125 | 0.883 | 0.001 |  |
| Temporal pole | 5.70 (0.08) | 5.71 (0.07) | 5.71 (0.08) | 2.013 | 0.135 | 0.009 |  |
| **Frontal lobe** | 5.68 (0.05) | 5.69 (0.05) | 5.68 (0.07) | 0.874 | 0.418 | 0.004 |  |
| Orbitofrontal | 5.79 (0.06) | 5.80 (0.05) | 5.79 (0.09) | 1.321 | 0.268 | 0.006 |  |
| Inferior frontal | 5.72 (0.06) | 5.72 (0.05) | 5.73 (0.06) | 1.646 | 0.194 | 0.007 |  |
| Middle frontal | 5.72 (0.06) | 5.73 (0.06) | 5.75 (0.06) | 10.763 | <0.001 | 0.046 | a, b < c |
| Superior frontal | 5.69 (0.08) | 5.70 (0.06) | 5.72 (0.07) | 7.087 | 0.001 | 0.031 | a < c |
| Precentral | 5.65 (0.06) | 5.65 (0.06) | 5.66 (0.07) | 2.296 | 0.102 | 0.010 |  |
| Paracentral | 5.72 (0.09) | 5.73 (0.09) | 5.74 (0.09) | 2.767 | 0.064 | 0.012 |  |
| Frontal pole | 5.60 (0.08) | 5.59 (0.08) | 5.61 (0.10) | 2.044 | 0.131 | 0.009 |  |
| Anterior cingulate | 5.64 (0.06) | 5.65 (0.07) | 5.66 (0.06) | 4.489 | 0.012 | 0.020 |  |
| **Parietal lobe** | 5.68 (0.04) | 5.68 (0.06) | 5.68 (0.05) | 0.217 | 0.805 | 0.001 |  |
| Inferior parietal | 5.71 (0.05) | 5.72 (0.05) | 5.72 (0.05) | 1.422 | 0.242 | 0.006 |  |
| Superior parietal | 5.77 (0.05) | 5.77 (0.05) | 5.78 (0.05) | 1.724 | 0.180 | 0.008 |  |
| Postcentral | 5.74 (0.05) | 5.75 (0.05) | 5.75 (0.05) | 2.170 | 0.115 | 0.010 |  |
| Precuneus | 5.74 (0.05) | 5.76 (0.05) | 5.76 (0.05) | 2.429 | 0.089 | 0.011 |  |
| Supra marginal | 5.66 (0.06) | 5.67 (0.05) | 5.68 (0.06) | 2.773 | 0.064 | 0.012 |  |
| Isthmus cingulate | 5.67 (0.05) | 5.66 (0.06) | 5.66 (0.06) | 0.217 | 0.805 | 0.001 |  |
| Posterior cingulate | 5.71 (0.05) | 5.72 (0.06) | 5.72 (0.05) | 2.483 | 0.085 | 0.011 |  |
| **Occipital lobe** | 5.86 (0.05) | 5.86 (0.04) | 5.86 (0.05) | 0.147 | 0.863 | 0.001 |  |
| Cuneus | 5.93 (0.07) | 5.93 (0.07) | 5.92 (0.07) | 2.718 | 0.067 | 0.012 |  |
| Lingual | 5.91 (0.05) | 5.92 (0.06) | 5.92 (0.05) | 1.757 | 0.174 | 0.008 |  |
| Lateral occipital | 5.86 (0.05) | 5.86 (0.04) | 5.85 (0.05) | 0.043 | 0.958 | 0.000 |  |
| Pericalcarine | 5.97 (0.08) | 5.96 (0.07) | 5.97 (0.07) | 0.715 | 0.490 | 0.003 |  |
| **Subcortical gray matter** | 5.44 (0.10) | 5.45 (0.07) | 5.45 (0.08) | 2.115 | 0.122 | 0.009 |  |
| Accumbens area | 5.40 (0.15) | 5.37 (0.15) | 5.35 (0.18) | 1.767 | 0.172 | 0.008 |  |
| Caudate | 5.42 (0.14) | 5.47 (0.11) | 5.50 (0.13) | 17.233 | <0.001 | 0.072 | a < b, c |
| Putamen | 5.69 (0.09) | 5.71 (0.10) | 5.74 (0.10) | 13.780 | <0.001 | 0.059 | a < b, c |
| Pallidum | 5.67 (0.14) | 5.66 (0.13) | 5.70 (0.11) | 3.729 | 0.025 | 0.017 | a < c |
| Thalamus | 5.30 (0.12) | 5.30 (0.10) | 5.32 (0.13) | 1.051 | 0.350 | 0.005 |  |
| **Cerebellum** | 5.49 (0.06) | 5.51 (0.06) | 5.52 (0.06) | 9.387 | <0.001 | 0.041 | a < c |

Note. All values are presented as mean (standard deviation)

N-CN, cognitively normal without neurodegeneration; N+CN, cognitively normal with neurodegeneration; N+MCI, mild cognitive impairment with neurodegeneration

***** One-way analysis of covariance adjusting for corresponding regional volume, age, sex, and education with Bonferroni-corrected post hoc comparisons

**Supplementary Table 5. Comparison of regional GLCM autocorrelation across diagnostic groups**

|  | N-CN^a^ | N+CN^b^ | N+MCI ^c^ | Statistics^*^ | | | |
| --- | --- | --- | --- | --- | --- | --- | --- |
|  | (n = 183) | (n = 111) | (n = 155) | F | *p* | effect size | post-hoc |
| **Temporal lobe** | 269.28 (3.14) | 270.01 (3.18) | 270.70 (3.27) | 5.284 | 0.005 | 0.023 | a < b, c |
| Insula | 267.19 (6.01) | 268.24 (4.81) | 269.00 (5.16) | 2.584 | 0.077 | 0.012 |  |
| Amygdala | 273.44 (12.03) | 276.14 (16.00) | 276.64 (12.88) | 2.594 | 0.076 | 0.012 |  |
| Hippocampus | 269.12 (5.97) | 274.40 (8.32) | 279.69 (15.55) | 26.247 | <0.001 | 0.106 | a,< b < c |
| Entorhinal cortex | 274.33 (9.21) | 275.91 (12.72) | 279.79 (14.60) | 3.031 | 0.049 | 0.014 | a < c |
| Parahippocampus | 268.82 (7.58) | 269.82 (7.40) | 271.48 (11.27) | 2.998 | 0.051 | 0.013 | a < c |
| Fusiform | 273.32 (5.04) | 274.11 (5.66) | 273.79 (4.18) | 0.370 | 0.691 | 0.002 |  |
| Bankssts | 268.01 (14.47) | 269.64 (8.71) | 268.13 (7.67) | 0.504 | 0.604 | 0.002 |  |
| Inferior temporal | 269.85 (6.71) | 270.48 (3.97) | 270.77 (3.92) | 0.475 | 0.622 | 0.002 |  |
| Middle temporal | 269.39 (4.73) | 270.52 (4.34) | 271.09 (3.71) | 4.829 | 0.008 | 0.021 | a < c |
| Superior temporal | 270.62 (3.87) | 271.27 (3.64) | 271.11 (3.55) | 0.886 | 0.421 | 0.004 |  |
| Transverse temporal | 342.26 (35.4) | 350.47 (35.82) | 351.97 (33.95) | 2.480 | 0.085 | 0.011 |  |
| Temporal pole | 273.24 (7.53) | 274.35 (6.57) | 275.76 (10.04) | 2.633 | 0.073 | 0.012 |  |
| **Frontal lobe** | 271.48 (2.74) | 271.82 (3.03) | 270.88 (3.83) | 2.625 | 0.074 | 0.012 |  |
| Orbitofrontal | 269.36 (2.79) | 270.56 (2.90) | 272.18 (5.62) | 19.057 | <0.001 | 0.079 | a < b, c |
| Inferior frontal | 273.47 (6.01) | 274.68 (4.40) | 275.04 (4.69) | 3.241 | 0.040 | 0.014 | a < c |
| Middle frontal | 269.93 (5.60) | 270.22 (3.39) | 271.23 (6.07) | 2.514 | 0.082 | 0.011 |  |
| Superior frontal | 273.13 (4.26) | 272.75 (3.97) | 272.27 (4.19) | 2.011 | 0.135 | 0.009 |  |
| Precentral | 280.07 (11.46) | 279.70 (8.80) | 279.87 (11.66) | 0.081 | 0.922 | 0.000 |  |
| Paracentral | 303.70 (24.30) | 302.23 (23.39) | 299.64 (22.90) | 1.599 | 0.203 | 0.007 |  |
| Frontal pole | 286.36 (14.49) | 288.72 (14.80) | 287.67 (15.37) | 0.552 | 0.576 | 0.002 |  |
| Anterior cingulate | 264.88 (7.67) | 265.02 (4.98) | 264.78 (4.88) | 0.122 | 0.885 | 0.001 |  |
| **Parietal lobe** | 273.34 (5.72) | 272.95 (3.22) | 272.82 (3.31) | 0.977 | 0.377 | 0.004 |  |
| Inferior parietal | 270.08 (8.21) | 270.38 (4.98) | 269.59 (3.70) | 0.493 | 0.611 | 0.002 |  |
| Superior parietal | 274.16 (10.27) | 273.41 (6.53) | 272.23 (5.03) | 2.457 | 0.087 | 0.011 |  |
| Postcentral | 277.03 (9.41) | 276.25 (6.25) | 276.39 (5.93) | 0.583 | 0.559 | 0.003 |  |
| Precuneus | 276.79 (10.79) | 276.22 (5.63) | 275.78 (5.33) | 1.072 | 0.343 | 0.005 |  |
| Supra marginal | 270.21 (6.52) | 270.99 (3.68) | 270.57 (3.81) | 0.280 | 0.756 | 0.001 |  |
| Isthmus cingulate | 264.91 (6.19) | 266.66 (7.09) | 267.87 (12.35) | 5.377 | 0.005 | 0.024 | a < c |
| Posterior cingulate | 268.25 (8.56) | 268.69 (6.40) | 267.47 (7.11) | 0.829 | 0.437 | 0.004 |  |
| **Occipital lobe** | 269.75 (9.22) | 268.89 (3.88) | 269.67 (6.49) | 0.575 | 0.563 | 0.003 |  |
| Cuneus | 297.04 (29.82) | 293.14 (22.43) | 290.61 (26.08) | 2.677 | 0.070 | 0.012 |  |
| Lingual | 280.27 (13.92) | 282.74 (14.12) | 282.00 (16.98) | 0.805 | 0.448 | 0.004 |  |
| Lateral occipital | 272.87 (12.41) | 271.29 (6.62) | 273.01 (7.70) | 1.889 | 0.152 | 0.008 |  |
| Pericalcarine | 312.43 (32.27) | 314.19 (26.52) | 311.34 (31.13) | 0.382 | 0.683 | 0.002 |  |
| **Subcortical gray matter** | 289.12 (16.33) | 290.06 (10.02) | 289.05 (9.59) | 0.291 | 0.748 | 0.001 |  |
| Accumbens area | 269.51 (29.42) | 283.81 (35.84) | 290.75 (36.81) | 14.016 | <0.001 | 0.060 | a < b, c |
| Caudate | 283.22 (14.48) | 284.15 (8.97) | 284.82 (12.33) | 0.551 | 0.577 | 0.002 |  |
| Putamen | 262.72 (6.36) | 263.55 (7.14) | 265.27 (9.87) | 3.562 | 0.029 | 0.016 | a < c |
| Pallidum | 290.63 (38.06) | 290.65 (33.22) | 286.09 (28.89) | 0.480 | 0.480 | 0.003 |  |
| Thalamus | 312.97 (29.03) | 311.88 (24.85) | 310.55 (21.47) | 0.667 | 0.514 | 0.003 |  |
| **Cerebellum** | 289.42 (14.36) | 286.44 (8.87) | 287.01 (9.22) | 2.736 | 0.066 | 0.012 |  |

Note. All values are presented as mean (standard deviation)

N-CN, cognitively normal without neurodegeneration; N+CN, cognitively normal with neurodegeneration; N+MCI, mild cognitive impairment with neurodegeneration

***** One-way analysis of covariance adjusting for corresponding regional volume, age, sex, and education with Bonferroni-corrected post hoc comparisons
